# Isoproterenol-Induced Cardiac Diastolic Dysfunction in Mice: A Systems Genetics Analysis

**DOI:** 10.3389/fcvm.2019.00100

**Published:** 2019-07-31

**Authors:** Jessica Wang, Adriana Huertas-Vazquez, Yibin Wang, Aldons J. Lusis

**Affiliations:** ^1^Department of Medicine, University of California, Los Angeles, Los Angeles, CA, United States; ^2^Department of Anesthesiology, University of California, Los Angeles, Los Angeles, CA, United States

**Keywords:** diastolic function, genome-wide association, isoproterenol, mouse, gene expression

## Abstract

We examined an isoproterenol heart failure model across a panel of diverse inbred strains of mice, the Hybrid Mouse Diversity Panel (HMDP), using left atrial (LA) and lung weights as well as echocardiogram parameters as surrogates for cardiac diastolic function. We identified gene transcripts that significantly correlated with diastolic function. In addition, we mapped echocardiographic parameters associated with diastolic function. We identified a locus near *Tns3-Hus1* to be associated with baseline E/A ratio in mice (*p* = 1.65E-06), the syntenic region of which was recently associated with E/A ratio in a genome-wide association study (GWAS) meta-analysis of the EchoGen consortium in humans. We also identified a locus near *Cdkn2a*-*Cdkn2b*, which is a region syntenic to the human 9p21 locus, to be associated with week 3 A/E ratio (*p* = 2.15E-06). Our study is the first study to map diastolic dysfunction in mice, in which a locus was found to be shared with a recent human GWAS on diastolic function. Moreover, our cardiac transcriptome correlation and eQTL analysis generated hypotheses for future basic investigations. These results showed that, although technical and physiological challenges limit diastolic function assessment in mice and humans, future investigations examining the genetic architecture of diastolic function among a diverse mouse population, such as the HMDP, in controlled experimental settings, offer distinct advantages in understanding the genetic determinants of diastolic function.

## Introduction

Heart failure with preserved ejection fraction (HFpEF) is a clinical syndrome that is characterized by diastolic dysfunction, involving impaired relaxation and compliance of the left ventricle (LV), leading to increased LV filling pressures, heart failure symptoms and an elevated risk of mortality (1–4). Among 5.7 million Americans with heart failure, HFpEF accounts for 50% of all heart failure hospital admissions (5). Although the understanding of HFpEF pathophysiology and risk factors, such as older age, female sex, hypertension, metabolic syndrome, renal dysfunction and obesity, has improved, treatment aimed at altering its natural history has yet to be developed due to the lack of understanding of its molecular underpinnings (6).

The Hybrid Mouse Diversity Panel (HMDP) consists of ~100 well-characterized inbred mouse strains that can be used to study genetic and environmental factors underlying complex traits (7). The HMDP, with a fully annotated genome and an easily controlled environment, can be readily manipulated to create a disease model for in-depth multi-omics molecular characterization at the tissue level. We had previously reported isoproterenol-induced cardiac phenotypes in the HMDP, in which we identified and validated a number of genomic loci associated with systolic heart failure traits via a genome-wide association approach (8–10). Chronic isoproterenol administration in mice has previously been demonstrated to induce diastolic dysfunction, as shown by an upward shift of the pressure-volume relationship (11). Although diastolic function can be assessed by directly measuring LV filling pressures via invasive hemodynamic monitoring (12), it can also be assessed by a number of echocardiographic findings non-invasively. These parameters, E and A velocities, tissue E' velocity, and their derivatives E/A ratio and E/E' ratio, have been used to study diastolic function in mice (13). The present study focuses on diastolic function data and analyses that have not been previously published. In this study we analyzed diastolic function parameters from the heart failure HMDP to identify candidate genomic loci and gene transcripts that may more accurately reflect the pathophysiology of diastolic dysfunction. Finally, we hypothesize the use of a diverse mouse population, such as the HMDP, to help us better understand diastolic dysfunction.

## Methods

### Assessment of Diastolic Function in Mice

Female mice at a median age of 9 weeks from 104 of the HMDP inbred strains were treated with isoproterenol (30 mg/kg/d) via an intra-abdominally implanted osmotic pump for 21 days as previously described (8–10). Briefly, baseline (prior to isoproterenol pump implantation), week 1, week 2, and week 3 echocardiograms were obtained. Due to tissue E' and A' velocities not being available on the VisualSonics Vevo 770 ultrasonography system used for this study, diastolic function was assessed by E and A velocities and via left atrial and lung weights. At the end of the protocol, heart chambers and organs were dissected and weighed. Left ventricular tissue was frozen in liquid nitrogen and later prepared for gene expression profiling as previously described (8–10).

### GWAS and Correlation Analysis

The HMDP mouse strains were previously genotyped using the JAX Mouse Diversity Genotyping Array (14). Mapping of diastolic function traits was performed using the Factored Spectrally Transformed Linear Mixed Models (FaST-LMM) as previously described (15). Gene-trait correlation analyses were performed using WGCNA (16). Additional details of the methods were previously described (8–10). The threshold for genome-wide significance in mice was previously determined through simulation and permutation of strain genotypes in the hybrid mouse diversity panel to be 4.1 × 10^−6^ by Farber et al. The significance threshold for local expression quantitative trait loci (eQTL) was similarly determined to be 3.63 × 10^−3^. In our exam of multiple related diastolic function traits, the genome-wide significance threshold for each clinical trait was kept at the nominal *p*-value of 4.1 × 10^−6^ without further Bonferroni adjustment to account for each trait tested. We applied the Benjamini-Hochberg (BH) procedure with a false discovery rate of 5% to account for multiple testing in trait-expression correlation analysis.

## Results

One-hundred and four classical and recombinant inbred mouse strains of the HMDP underwent intraperitoneal implantation of isoproterenol mini-pumps. Global gene expression of the left ventricular tissue was performed as previously reported (8–10). Diastolic function was assessed by echocardiographic measures, such as E and A velocities, and left atrial and lung weights, which reflect cumulative impact of isoproterenol on LV filling pressures over time, leading to atrial volume increase, pulmonary congestion, and edema (17). Tissue velocity measurement was not available on the ultrasonography system used for the study.

### Diastolic Function Assessment Across the HMDP Population

**Figure 1:**
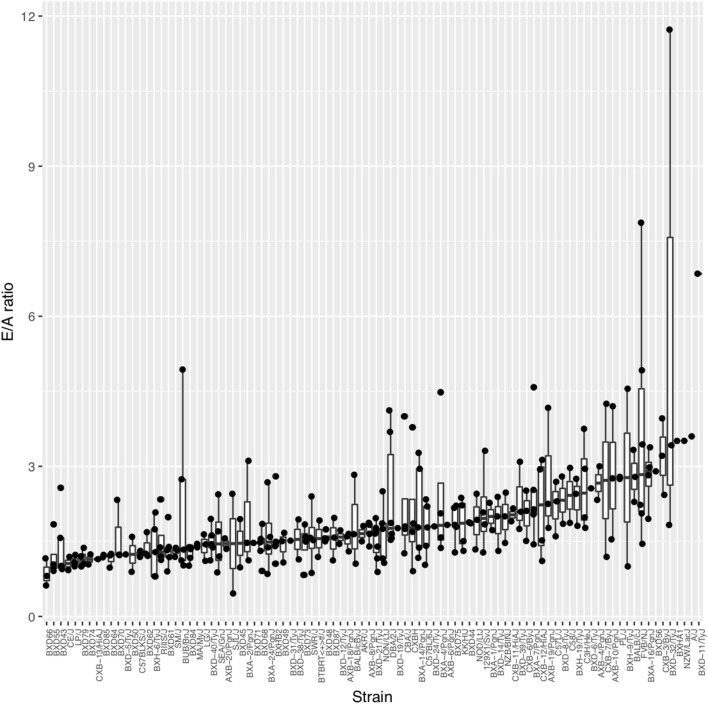
The spectrum of diastolic function as measured by E/A ratio across the Hybrid Mouse Diversity Panel (HMDP) strains. Boxplots represent the intra-strain variation in E/A ratio. Strains are ordered by median E/A ratio.

On average across the HMDP strains, left atrial weight at the time of sacrifice had increased by 78.8% among isoproterenol treated vs. control mice (4.29 ± 1.69 mg vs. 2.4 ± 0.583 mg; *p* = 9.33E-20), indicating significant left atrial remodeling due to isoproterenol-induced elevated filling pressures over 3 weeks (Supplementary Table 1A). Of note, ejection fraction (EF) did not change on average between baseline and week 3 of isoproterenol. These findings are consistent with an isoproterenol-induced HFpEF model on average among HMDP mice. In terms of echocardiographic measures of diastolic function, average E and A velocities increased with isoproterenol during the first week and, thereby, remained relatively unchanged for the rest of isoproterenol treatment (Supplementary Table 2). E/A ratio did not change significantly on average (Supplementary Table 1B). There was a wide distribution of E/A ratio among HMDP strains at week 3 of isoproterenol with only a minority of strains with E/A ratio < 1, indicating varying degrees of diastolic dysfunction (Figure 1). There is no significant correlation between E/A and fibrosis (Supplementary Figure 1), indicative of intrinsic relaxation properties rather than extracellular fibrotic processes as the primary driver of the E/A ratio phenotype.

### Diastolic Function Correlation With Left Ventricular Gene Transcripts

We examined isoproterenol-treated transcripts that were most significantly correlated with diastolic function traits such as left atrial weight, lung weight, E and A velocities (Table 1). Many of the significantly correlated gene transcripts have been reported to be expressed in the heart but their specific roles in heart failure have not been previously explored. A few of the notable gene transcripts with known roles in the heart are described as follows. *Adamts2*, which was significantly correlated with lung weight (*r* = 0.459, *p* = 5.49e−06), has been previously reported by our group to be a driver of cardiac hypertrophy (8) and confirmed by another group using gain- and loss-of-function genetic mouse model of *Adamts2* to negatively regulate cardiomyocyte hypertrophy via the PI3K/AKT-dependent signaling pathway (18). *Vav2*, which was correlated with E velocity at week 3 (*r* = 0.469, *p* = 4.12e−06), is a signal transduction molecule that activates Rho/Rac GTPases, the knockout of which in mice causes tachycardia, hypertension, defects in the heart, arterial walls and kidneys via chronic stimulation of the renin/angiotensin II and sympathetic nervous systems (19). *Tmod4*, a muscle-specific member of the tropomodulin family of actin-regulatory proteins (20), was found to be negatively correlated with left atrial weight (*r* = −0.466, *p* = 3.69e−06). Moreover, *Jmjd1a*, a histone 3 lysine 9 (H3K9)-specific demethylase that binds all 3 myocardin family members, was positively correlated with A velocity at week 3 with borderline significance (*r* = 0.457, *p* = 7.69e−06, BH adjusted *p* = 0.0549) and is known to be upregulated in myocardium from patients with hypertrophic heart disease (21). Finally, *Col6a1*, which was correlated with lung weight with borderline significance (*r* = 0.451, *p* = 7.95e−06, BH adjusted *p* = 0.0549) has previously been shown, when absent, to be protective following myocardial infarction by limiting infarct size, apoptosis, aberrant remodeling and fibrosis (22).

**Table 1:** Significantly correlated isoproterenol-induced transcripts with diastolic function parameters including left atrial weight, lung, E and A velocity and E/A ratio.

	**Gene symbol**	**Probe ID**	**Phenotype**	**Pearson correlation**	***P*-value**	**Adjusted *p*-value**
1	Stx16	ILMN_2735822	E velocity	0.513	3.26E-07	0.019
2	Ms4a4d	ILMN_2735547	E velocity	0.500	7.13E-07	0.025
3	Vav2	ILMN_1223067	E velocity	0.469	4.12E-06	0.044
4	Galr3	ILMN_2887408	LA weight	0.561	9.08E-09	0.005
5	Olfr1306	ILMN_3162277	LA weight	0.526	1.01E-07	0.013
6	Nrip3	ILMN_2875404	LA weight	0.516	2.00E-07	0.017
7	Olfr803	ILMN_2718049	LA weight	0.508	3.19E-07	0.019
8	Lmo3	ILMN_3139548	LA weight	0.505	3.86E-07	0.019
9	Gzmm	ILMN_1229500	LA weight	−0.495	6.99E-07	0.025
10	Ndufa1	ILMN_1251370	LA weight	−0.492	8.62E-07	0.027
11	Chrnd	ILMN_2662184	LA weight	0.483	1.39E-06	0.030
12	March4	ILMN_2793827	LA weight	0.478	1.85E-06	0.035
13	Rab6ip1	ILMN_2635232	LA weight	0.475	2.30E-06	0.038
14	2310044D20Rik	ILMN_2742739	LA weight	0.473	2.45E-06	0.038
15	Nbn	ILMN_2880745	LA weight	0.468	3.38E-06	0.043
16	Rab6ip1	ILMN_3107607	LA weight	0.466	3.64E-06	0.043
17	Tmod4	ILMN_2488846	LA weight	−0.466	3.69E-06	0.043
18	Whrn	ILMN_3082031	LA weight	−0.465	3.93E-06	0.044
19	1700001E04Rik	ILMN_2724055	LA weight	0.464	4.19E-06	0.044
20	4921505C17Rik	ILMN_2694387	LA weight	0.461	4.77E-06	0.044
21	Umps	ILMN_1254813	LA weight	0.456	6.15E-06	0.048
22	Gzmm	ILMN_1229500	Lung weight	−0.502	4.50E-07	0.020
23	Olfr1306	ILMN_3162277	Lung weight	0.490	9.53E-07	0.028
24	1110032E23Rik	ILMN_1235811	Lung weight	0.488	1.08E-06	0.029
25	Rab6ip1	ILMN_3107607	Lung weight	0.484	1.35E-06	0.030
26	Ube2e2	ILMN_2792485	Lung weight	0.463	4.34E-06	0.044
27	Rab6ip1	ILMN_2635232	Lung weight	0.462	4.55E-06	0.044
28	Adamts2	ILMN_1226259	Lung weight	0.459	5.49E-06	0.047
29	Srpx2	ILMN_2818294	Lung weight	0.457	5.93E-06	0.048

*The Benjamini-Hochberg procedure with a false discovery rate of 5% was applied to account for multiple testing*.

### Genome-Wide Association of Diastolic Function Parameters Across the HMDP Population

**Figure 2:**
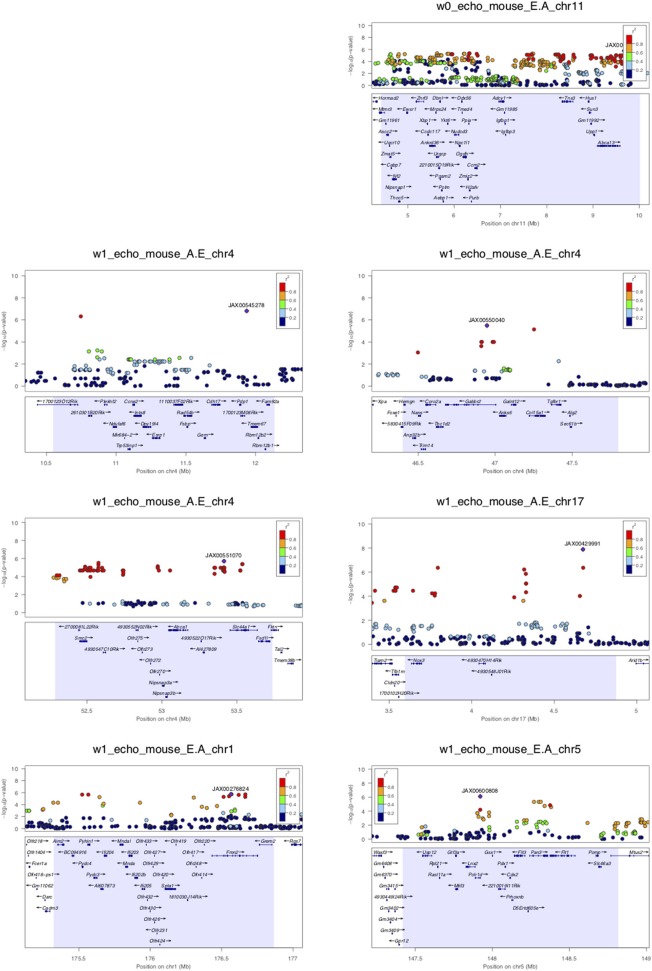
LocusZoom plots for regional visualization of genome-wide association scan results of echocardiographic diastolic function loci. The regional lead single nucleotide polymorphism (SNP) is represented by the purple diamond symbol and annotated by its SNP ID. Surrounding SNPs are color coded according to the strength of pairwise linkage disequilibrium (LD) in terms of the r^2^ metric to the lead SNP. w0, w1, w2, and w3 denote baseline, week 1, week 2, and week 3 of isoproterenol. E.A denotes E/A ratio. A.E denotes A/E ratio.

Assuming that diastolic function cannot be normal under continuous beta-adrenergic stimulation by isoproterenol over 3 weeks, E/A ratio measurements may be treated as a monotonic continuous variable, where the higher the value the more diastolic dysfunction there is. This assumption obviates the need to distinguish normal vs. pseudonormal (grade II) patterns. We mapped E/A ratio and its inverse A/E ratio (in case the inverse value of the E/A ratio performs better on the linear mixed model utilized by FaST-LMM for association testing) across the HMDP on isoproterenol and identified 11 significant loci for diastolic function (Figure 2 and Table 2). Mapping of left atrial weight did not yield significant loci, which could reflect isoproterenol driving the phenotype to the extreme such that there is insufficient variation to map. Here we highlight a few of the more interesting loci.

**Table 2:** Significant echocardiographic diastolic function loci.

**Analysis**	**Trait**	**Lead SNP**	***P*-value**	**MAF**	**Genomic location of lead SNP (chr:pos)**	**Lead SNP type**	**Locus (Mb)**	**Candidate gene(s)**	**Exp**	**eQTL**	**Cor**	**SV**
Week 0	E/A	rs29436095	1.65E-06	0.32	11:9672375	Intergenic	4.4–10.1	*Tns3* *Hus1* *Sun3* *Upp1* *Abca13*	– 8_d_ – 8 4	– Yes – Yes No	– Yes – No No	M 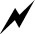 – – – MS
Week 1	E/A	rs33802297 rs33797952	1.86E-06	0.13	1:176565276 -176569258	Intronic	175.3–176.9	*Fmn2* 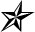	4	No	No	S
Week 1	E/A	rs32376064	7.82E-07	0.30	5:147921722	Intronic	147.4–148.9	*Lnx2* *Polr1d* 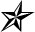 *Gsx1* *Flt3* *Pan3* *Flt1*	8_d_ – – 4 – 11	Yes – – No – No	No – – No – Yes	– – – – – –
Week 2	E/A	rs29910955	2.69E-06	0.34	13:75682216	Intergenic	72.1–76.1	*Hnrnpa1l2-ps* *Pcsk1* *Gm4149* *Mir682* *Ell2* *Glrx* *Rhobtb3* *Gm15528* *Gm6311* *Spata9* *Rfesd*	– – – – 4 11^d^ 9 – – – 5	– – – – No No No – – – No	– – – – No Yes No – – – No	– – – – – – – – – – –
Week 3	E/A	rs32625361	3.13E-07	0.39	4:96577960	Intergenic	96.3–98.1	*E130114P18Rik* *Nfia* *0610025J13Rik* *Tm2d1*	– 4 – –	– No – –	– No – –	– – – –
Week 1	A/E	rs27746189	1.54E-07	0.20	4:11936178	Exonic	10.5–12.2	*AK076817* 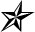	–	–	–	
Week 1	A/E	rs27821865	3.25E-06	0.28	4:46946779	Intronic	45.6–48.9	*Gabbr2* 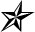	–	–	–	
Week 1	A/E	rs27794930	1.96E-06	0.25	4:53413255	Intergenic	52.2–53.8	*Smc2* *Nipsnap3b* *Abca1* *Slc44a1*	– 6^d^ 4 6	– Yes No No	– Yes No Yes	MS – – S
Week 1	A/E	rs33594278	1.29E-08	0.30	17:4674140	Intronic	3.2–4.9	*Tiam2* *Tfb1m* *Cldn20* *Nox3* *Arid1b* *AK186662* 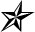	5 – – 4 – –	No – – No – –	No – – No – –	– – – – – –
Week 3	A/E	rs28160199	2.15E-06	0.33	4:89040388	Intronic	88.3–89.8	*Mtap* *Cdkn2a* *Cdkn2b* *AK148321* 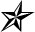 *Dmrta1*	8 4 – – 4	No No – – No	No No – – No	M – – – –
Week 3	A/E	rs28111681 rs28096652 rs28096421	9.20E-07	0.43	4:97293373-97339169	Intronic	96.3–98.1	*Nfia* 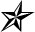	4	No	No	–

*MAF denotes minor allele frequency. Locus denotes the region surrounding the lead SNP where regional SNPs are in r^2^ > 0.8 linkage disequilibrium (LD) with the lead SNP. Candidate genes are short listed by LD cutoffs of r^2^ > 0.9 or 0.95, if applicable. 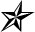 denotes the lead SNP is either intronic or exonic to the annotated gene. – Data is not available. Exp denotes approximate average log_2_ intensity of expression in the left ventricule, annotated by ^d^for up- and _d_for down-regulation by isoproterenol. eQTL denotes the presence of cis-eQTL (p-value < 3.63 × 10^−3^) in the left ventricle. Cor denotes p < 0.05 for Pearson correlation between gene expression and clinical trait. SV denotes presence of structural variations among the classical inbred strains, including M for missence where SIFT score is predicted to be deleterious, S for splice region variants and 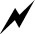 for premature stop*.

Week 0 E/A ratio mapped to a linkage disequilibrium block (*r*^2^ > 0.9) around SNP rs29436095 spanning 5 genes. Of note, in a large genome-wide association meta-analysis of studies in the EchoGen consortium, E/A ratio was mapped to a statistically suggestive human locus near *Tns3* (*p* = 2.11e−4), which is syntenic to this region. The AA and GG genotypes at lead SNP rs29436095 conferred a median E/A ratio of 1.66 and 2.11, respectively. Among these genes, *Hus1* expression was downregulated with isoproterenol. *Hus1* expression under the control condition showed a trend to positive correlation with E/A ratio (cor = 0.193, *p* = 0.0867) and mapped to the same locus as E/A ratio, indicative of a cis-eQTL that may underly the phenotype of week 0 E/A ratio. These results suggest that *Hus1* may play a role in baseline left ventricular diastolic function and is regulated with chronic isoproterenol administration.

Week 1 A/E ratio mapped to a locus near SNP rs27794930, which is in an intergenic region between *Abca1* and *Slc44a1*. Nearby SNPs in *r*^2^ > 0.95 LD with the lead SNP spanned a ~1.5 Mb window containing many genes. Among these genes, *Nipsnap3b* expression was upregulated with isoproterenol. *Nipsnap3b* expression under the control condition was positively correlated with week 1 A/E ratio (ILMN_2657376, cor = 0.32, *p* = 0.004; ILMN_2592629, cor = 0.25, *p* = 0.02) and mapped to the same locus as A/E ratio, indicative of a cis-eQTL. Our results suggest that *Nipsnap3b* may play a role in left ventricular diastolic function at week 1 of isoproterenol and is regulated by chronic isoproterenol administration. *Nipsnap3b*, which is highly expressed in skeletal muscle and heart, is a member of the NIPSNAP family with putative roles in vesicular trafficking (23, 24).

Week 3 A/E ratio and E/A ratio mapped to a locus near *Nfia* (Figure 2B). Although there are currently no follow up data regarding the role of nuclear factor 1 a in the heart, this locus has previously been associated with QRS duration on ECG, arrhythmia, and cardiomyopathy (25). Finally, week 3 A/E ratio also mapped to a locus near SNP rs28160199, which is in an intronic region of a non-coding RNA *AK148321*. Nearby SNPs in *r*^2^ > 0.8 LD with the lead SNP spanned across a region syntenic to the human 9p21 locus, containing *ANRIL, CDKN2A*, and *CDKN2B*, that has been associated with coronary artery disease, diabetes, and multiple cancers (26, 27).

## Discussion

This is the first study to genetically map diastolic function phenotypes in mice, in which a locus was found to be shared with a recent human GWAS on diastolic function. Moreover, we presented cardiac transcriptome correlation and cis-eQTL analyses that generated hypotheses regarding the involvement of the presented candidate genes and loci in the pathophysiology of diastolic dysfunction to be confirmed in follow-up basic investigations.

Recently, a large GWAS meta-analysis of echocardiographic traits, that included 46,533 individuals from 30 studies of the EchoGen consortium, evaluated 16 traits encompassing left ventricular structure, systolic and diastolic function and revealed a single significantly associated locus (28). This locus flagged by a lead intronic SNP rs12440869 on chromosome 15 near gene IQCH was found to be significantly associated with transmitral A-wave peak velocity (meta-analysis *p* = 1.31e-08). In addition, twenty-three suggestive loci for transmitral E-wave velocity, E/A ratio, deceleration time, isovolumetric relaxation time (IVR), E' velocity, E/E' velocity, diastolic dysfunction with preserved ejection function, and heart failure with preserved ejection function were reported (28). Molecular mechanisms leading to diastolic dysfunction underlying these human loci have yet to be confirmed by basic investigations. Of interest, one of the suggestive loci near *Tns3* is syntenic to a locus that week 0 E/A ratio mapped to in our study. The shared locus identified for E/A ratio suggests that a systems genetics approach in mice could be a feasible alternative in dissecting the genetic architecture of diastolic pathophysiology. Moreover, the *Tns3* locus contains many genes. We were able to prioritize *Hus1* as a putative causal gene based on cardiac tissue transcriptome correlation and cis-eQTL analyses, highlighting another benefit of studying diastolic function using the systems genetics approach in mice.

Although widely accepted in clinical practice and previously reported to accurately quantify the degree of cardiac diastolic performance in mice (13), there are a number of limitations and technical considerations worth noting regarding non-invasive assessment of diastolic function in both humans and mice. An important limitation of diastolic function assessment in mice is the difficulty in obtaining a true apical view to accurately measure mitral annular velocities by tissue Doppler. Also, diastolic function assessment in mice is somewhat complicated by a normal resting mouse heart rates at around 600 beats per minute. In the setting of excess rise, that is, heart rate > 650 bpm, due to stress of activation of the autonomic nervous system, fusion of LV filling early E and late A waves often occurs, making it difficult to assess diastolic function in mice (29). In a study examining diastolic function parameters using multiple mouse models of heart disease, mitral E/A ratio assessment was noted to be technically difficult due to high heart rates; combined measurement of left atrial area and reverse longitudinal strain rate and/or isovolumic relaxation time (IVRT) was felt to be necessary for an overall assessment of diastolic function (30). A major limitation of our study is that, at the time of our echocardiographic assessment, tissue Doppler velocity and longitudinal strain rate were not yet available. We relied on the ratio of mitral inflow E and A velocities, which is invariant to Doppler interrogation angle, as our primary echocardiographic measure of diastolic function for genome mapping. Thus, future diastolic assessment incorporating additional measures may more accurately capture the phenotype of interest.

Finally, diastolic function is a product of environmental, genetic, and co-morbid factors in humans and non-invasive parameters of diastolic function assessment are subjected to changes in loading conditions that may be difficult to control in both humans and mice, even under well-defined experimental settings. Quantitative measures of diastolic function that reflect intrinsic elastance, such as end diastolic pressure-volume relationship (EDPVR) and end systolic pressure-volume relationship (ESPVR) derived from invasive hemodynamic monitoring under different loading conditions, are robust and may significantly increase mapping power and accuracy. We hypothesize that genetic mapping of invasive measurements of left ventricular intrinsic elastance in a genetically diverse population, such as the HMDP, will significantly improve the accuracy of mapping for various models of diastolic dysfunction, such as aging and obesity, and accelerate the understanding of diastolic pathophysiology in mice and humans.

## Data Availability

Publicly available datasets were analyzed in this study. This data can be found here: https://www.ncbi.nlm.nih.gov/geo/query/acc.cgi?acc=GSE48760.

## Ethics Statement

All animal experiments were conducted following guidelines established and approved by the University of California, Los Angeles Institutional Animal Care and Use Committee. This study was carried out in accordance with the recommendations of the University of California, Los Angeles Institutional Animal Care and Use Committee. The protocol was approved by the University of California, Los Angeles Institutional Animal Care and Use Committee.

## Author Contributions

JW, YW, and AL contributed to conception and design of the study. JW performed echocardiograms, measured diastolic parameters, prepared samples for RNA isolation, organized the data, performed statistical analysis, and drafted the manuscript. AH-V provided critical revisions for important intellectual content of the manuscript. YW and AL provided critical feedback and mentorship. All authors contributed to manuscript revision and approved the submitted version.

### Conflict of Interest Statement

The authors declare that the research was conducted in the absence of any commercial or financial relationships that could be construed as a potential conflict of interest.
