# Supplementary material for: Isoproterenol-Induced Cardiac Diastolic Dysfunction in Mice: A Systems Genetics Analysis
Source: Front Cardiovasc Med. 2019 Jul 31;6:100. doi: 10.3389/fcvm.2019.00100 (PMC6684968; doi:10.3389/fcvm.2019.00100)
Supplement: Supplementary file 1 [file Data_Sheet_1.docx]

Supplementary Material

Supplementary Table 1. General Weight and Echocardiographic Characteristics at Baseline and at Week 3 of Isoproterenol. BW = body weight. TH = total heart weight. LV = left ventricular weight. RV = right ventricular weight. LA = left atrial weight. RA = right atrial weight. Lung = lung weight. Liver = liver weight. Adrenal = total adrenal weight. IVSd = interventricular septal wall thickness at end diastole. LVIDd = left ventricular internal diameter at end diastole. PWd = posterior wall thickness at end diastole. IVSs = interventricular septal wall thickness at end systole. LVIDs = left ventricular internal diameter at end systole. PWs = posterior wall thickness at end systole. ET = ejection time. E = early mitral inflow velocity. A = late mitral inflow velocity. E/A = E-to-A ratio. A/E = A-to-E ratio. FS = fractional shortening. IVS/PWd = interventricular septal wall thickness to posterior wall thickness ratio at end diastole. IVS/PWs = interventricular septal wall thickness to posterior wall thickness ratio at end systole. RWTd = relative wall thickness at end diastole. RWTd = relative wall thickness at end diastole. PWTH = posterior wall thickening. Vold = left ventricular volume at end diastole. Vols = left ventricular volume at end systole. EF = ejection fraction. LVM = left ventricular mass. Vcf = velocity of circumferential fiber shortening. MNSER = mean normalized systolic ejection rate.

1. Weight Characteristics (n=92 strains)

|  | ctrl mean | ctrl sd | iso mean | iso sd | % change | paired t-test p-value |
| --- | --- | --- | --- | --- | --- | --- |
| BW (g) | 22.4 | 3.41 | 23.6 | 3.17 | 5.16% | 4.33E-05 |
| TH (mg) | 98.9 | 13.9 | 133 | 24.2 | 34.50% | 7.71E-34 |
| LV (mg) | 68.9 | 10.6 | 92.3 | 16.6 | 33.90% | 1.55E-32 |
| RV (mg) | 12.3 | 2.73 | 15.2 | 4.25 | 22.90% | 1.63E-13 |
| **LA (mg)** | **2.4** | **0.583** | **4.29** | **1.69** | **78.80%** | **9.33E-20** |
| RA (mg) | 2.05 | 0.588 | 3.01 | 1.17 | 47.10% | 1.61E-12 |
| Lung (mg) | 127 | 16.1 | 154 | 29.4 | 21.60% | 7.19E-22 |
| Liver (mg) | 936 | 181 | 1040 | 196 | 11% | 1.95E-13 |
| Adrenal (mg) | 10.9 | 4.4 | 9.12 | 2.36 | -16.50% | 1.32E-06 |

B. Echocardiographic Characteristics (n=68 strains)

|  | ctrl mean | ctrl sd | iso mean | iso sd | % change | paired t-test p-value |
| --- | --- | --- | --- | --- | --- | --- |
| Heart Rate | 505 | 56.7 | 560 | 47.1 | 11.10% | 8.77E-12 |
| IVSd | 0.758 | 0.0924 | 0.805 | 0.0993 | 7.28% | 0.000837 |
| LVIDd | 3.66 | 0.337 | 4.13 | 0.342 | 12.40% | 1.14E-14 |
| PWd | 0.761 | 0.0966 | 0.804 | 0.097 | 6.39% | 0.00122 |
| IVSs | 1.15 | 0.151 | 1.21 | 0.183 | 6.71% | 0.00174 |
| LVIDs | 2.25 | 0.403 | 2.59 | 0.464 | 13.50% | 8.81E-06 |
| PWs | 1.08 | 0.142 | 1.16 | 0.162 | 8.14% | 0.000252 |
| ET | 50.4 | 5.03 | 45.4 | 2.96 | -10% | 2.06E-12 |
| **E** | **1670** | **499** | **1720** | **481** | **9.11%** | **0.0424** |
| **A** | **998** | **374** | **1060** | **442** | **17.10%** | **0.0138** |
| **E/A** | **1.91** | **0.911** | **1.98** | **0.895** | **0.71%** | **0.921** |
| A/E | 0.616 | 0.184 | 0.631 | 0.199 | 5.44% | 0.205 |
| FS | 39 | 6.55 | 38 | 7.51 | -1.07% | 0.69 |
| IVS/PWd | 1.01 | 0.11 | 1.02 | 0.105 | 0.75% | 0.655 |
| IVS/PWs | 1.08 | 0.129 | 1.06 | 0.0947 | -1.58% | 0.362 |
| RWTd | 0.422 | 0.0792 | 0.395 | 0.0621 | -5.43% | 0.0583 |
| PWTH | 42.7 | 14.4 | 45.7 | 15 | 5.26% | 0.255 |
| Vold | 57.8 | 12.8 | 76.9 | 15.3 | 31.60% | 3.22E-14 |
| Vols | 18.6 | 7.94 | 26.3 | 11.2 | 37.10% | 5.42E-06 |
| EF | 69.3 | 8.05 | 67.3 | 9.42 | -1.61% | 0.387 |
| LVM | 94.5 | 14.7 | 126 | 24.9 | 33.20% | 2.68E-21 |
| Vcf | 0.786 | 0.174 | 0.848 | 0.199 | 9.82% | 0.00952 |
| MNSER | 1.4 | 0.241 | 1.5 | 0.261 | 9.05% | 0.00164 |

Supplementary Table 2. Comparison of Echocardiographic Characteristics Between Weekly Intervals

A. Comparison of Echocardiographic Characteristics Between Baseline and Week 1 of Isoproterenol

|  | baseline mean | baseline sd | week 1 mean | week 1 sd | % change | paired t-test p-value |
| --- | --- | --- | --- | --- | --- | --- |
| BW | 21.1 | 3.03 | 20.8 | 3 | -2.49% | 0.00315 |
| Heart Rate | 481 | 39.6 | 573 | 55.4 | 18.80% | 1.90E-25 |
| IVSd | 0.725 | 0.0763 | 0.852 | 0.115 | 17.60% | 1.18E-19 |
| LVIDd | 3.71 | 0.233 | 3.91 | 0.353 | 4.95% | 8.98E-09 |
| PWd | 0.706 | 0.0707 | 0.823 | 0.106 | 16% | 6.13E-16 |
| IVSs | 1.04 | 0.121 | 1.28 | 0.201 | 23.40% | 3.55E-20 |
| LVIDs | 2.38 | 0.264 | 2.33 | 0.426 | -2.62% | 0.186 |
| PWs | 1.02 | 0.0915 | 1.22 | 0.176 | 19.50% | 7.34E-17 |
| ET | 52.3 | 3.75 | 44.4 | 3.91 | -14.70% | 1.43E-30 |
| E | 1530 | 459 | 1790 | 537 | 15.60% | 0.00026 |
| A | 866 | 297 | 1080 | 418 | 26.60% | 1.94E-06 |
| E/A | 1.99 | 0.586 | 1.95 | 0.621 | -4.76% | 0.245 |
| A/E | 0.575 | 0.135 | 0.607 | 0.164 | 7.74% | 0.0199 |
| FS | 36.5 | 5.03 | 40.9 | 6.96 | 12.70% | 2.24E-06 |
| IVS/PWd | 1.04 | 0.119 | 1.05 | 0.101 | 0.79% | 0.603 |
| IVS/PWs | 1.03 | 0.0985 | 1.07 | 0.117 | 3.37% | 0.0171 |
| RWTd | 0.383 | 0.044 | 0.429 | 0.0824 | 11.50% | 5.19E-07 |
| PWTH | 45.5 | 10.5 | 48.9 | 13.4 | 9% | 0.011 |
| Vold | 59.5 | 8.69 | 67.4 | 14.5 | 12.70% | 8.07E-09 |
| Vols | 20.5 | 5.68 | 20.4 | 9.27 | -2.32% | 0.648 |
| EF | 66.3 | 6.47 | 71.3 | 8.23 | 8.05% | 8.25E-06 |
| LVM | 89.9 | 13.6 | 121 | 23 | 33.60% | 4.32E-27 |
| Vcf | 0.707 | 0.113 | 0.942 | 0.201 | 33.90% | 1.63E-15 |
| MNSER | 1.28 | 0.161 | 1.64 | 0.263 | 28% | 3.75E-18 |

B. Comparison of Echocardiographic Characteristics Between Week 1 and Week 2 of Isoproterenol

|  | week 1 mean | week 1 sd | week 2 mean | week 2 sd | % change | paired t-test p-value |
| --- | --- | --- | --- | --- | --- | --- |
| BW | 20.8 | 3 | 22.6 | 2.94 | 7.60% | 2.97E-23 |
| Heart Rate | 573 | 55.4 | 551 | 56.5 | -4.42% | 0.000394 |
| IVSd | 0.852 | 0.115 | 0.801 | 0.109 | -5.96% | 0.000541 |
| LVIDd | 3.91 | 0.353 | 4.13 | 0.305 | 5.86% | 9.04E-14 |
| PWd | 0.823 | 0.106 | 0.795 | 0.11 | -3.53% | 0.0128 |
| IVSs | 1.28 | 0.201 | 1.2 | 0.187 | -7.21% | 7.45E-05 |
| LVIDs | 2.33 | 0.426 | 2.61 | 0.437 | 13.10% | 8.44E-11 |
| PWs | 1.22 | 0.176 | 1.14 | 0.17 | -6.63% | 0.000289 |
| ET | 44.4 | 3.91 | 46.5 | 5.09 | 5.29% | 0.000348 |
| E | 1790 | 537 | 1800 | 524 | -0.28% | 0.929 |
| A | 1080 | 418 | 1030 | 372 | -6.19% | 0.179 |
| E/A | 1.95 | 0.621 | 2.08 | 0.844 | 7.91% | 0.128 |
| A/E | 0.607 | 0.164 | 0.594 | 0.18 | -2.41% | 0.518 |
| FS | 40.9 | 6.96 | 37.3 | 6.98 | -9.71% | 2.50E-06 |
| IVS/PWd | 1.05 | 0.101 | 1.02 | 0.106 | -2.04% | 0.179 |
| IVS/PWs | 1.07 | 0.117 | 1.05 | 0.114 | -1.25% | 0.396 |
| RWTd | 0.429 | 0.0824 | 0.39 | 0.0684 | -9.15% | 8.60E-06 |
| PWTH | 48.9 | 13.4 | 45.1 | 12.3 | -8.23% | 0.0135 |
| Vold | 67.4 | 14.5 | 76.5 | 13.9 | 14.20% | 2.67E-13 |
| Vols | 20.4 | 9.27 | 26.6 | 11.3 | 33.40% | 4.33E-10 |
| EF | 71.3 | 8.23 | 66.6 | 9.48 | -7.18% | 1.44E-06 |
| LVM | 121 | 23 | 124 | 23.5 | 3.12% | 0.0315 |
| Vcf | 0.942 | 0.201 | 0.818 | 0.192 | -13% | 4.22E-06 |
| MNSER | 1.64 | 0.263 | 1.46 | 0.281 | -10.70% | 2.59E-06 |

C. Comparison of Echocardiographic Characteristics Between Week 2 and Week 3 of Isoproterenol

|  | week 2 mean | week 2 sd | week 3 mean | week 3 sd | % change | paired t-test p-value |
| --- | --- | --- | --- | --- | --- | --- |
| BW | 22.6 | 2.94 | 23.6 | 3.15 | 3.51% | 9.20E-15 |
| Heart Rate | 551 | 56.5 | 559 | 46.9 | 0.87% | 0.305 |
| IVSd | 0.801 | 0.109 | 0.803 | 0.101 | -0.21% | 0.866 |
| LVIDd | 4.13 | 0.305 | 4.15 | 0.358 | 0.22% | 0.702 |
| PWd | 0.795 | 0.11 | 0.802 | 0.0973 | 0.59% | 0.652 |
| IVSs | 1.2 | 0.187 | 1.2 | 0.189 | -0.64% | 0.668 |
| LVIDs | 2.61 | 0.437 | 2.61 | 0.502 | 0.62% | 0.646 |
| PWs | 1.14 | 0.17 | 1.16 | 0.165 | 0.20% | 0.892 |
| ET | 46.5 | 5.09 | 45.4 | 2.95 | -1.66% | 0.0402 |
| E | 1800 | 524 | 1780 | 662 | 0.58% | 0.856 |
| A | 1030 | 372 | 1080 | 446 | 4.31% | 0.327 |
| E/A | 2.08 | 0.844 | 1.99 | 0.899 | -2.70% | 0.55 |
| A/E | 0.594 | 0.18 | 0.627 | 0.2 | 3.80% | 0.335 |
| FS | 37.3 | 6.98 | 37.6 | 7.79 | -0.37% | 0.829 |
| IVS/PWd | 1.02 | 0.106 | 1.02 | 0.104 | -0.78% | 0.591 |
| IVS/PWs | 1.05 | 0.114 | 1.05 | 0.0952 | -0.28% | 0.845 |
| RWTd | 0.39 | 0.0684 | 0.394 | 0.0617 | 1.02% | 0.539 |
| PWTH | 45.1 | 12.3 | 45.3 | 15 | -2.77% | 0.498 |
| Vold | 76.5 | 13.9 | 77.5 | 15.8 | 0.67% | 0.616 |
| Vols | 26.6 | 11.3 | 27.1 | 12.3 | 3.54% | 0.289 |
| EF | 66.6 | 9.48 | 66.9 | 9.95 | -0.70% | 0.574 |
| LVM | 124 | 23.5 | 127 | 25.7 | 1.13% | 0.389 |
| Vcf | 0.818 | 0.192 | 0.841 | 0.203 | 1.25% | 0.568 |
| MNSER | 1.46 | 0.281 | 1.49 | 0.268 | 0.76% | 0.661 |

Supplementary Figure 1. Correlation between week 3 E/A ratio and fibrosis. Fibrosis was previously quantified in Rau et al. by comparing the amount of tissue stained blue for collagen upon Masson’s trichrome staining to the total tissue area.

Pearson corr = 0.21

p-value = 0.095

Fibrosis (a.u.)

E/A ratio
